# Investigating Countermovement and Horizontal Jump Asymmetry in Female Football Players: Differences Across Age Categories

**DOI:** 10.3390/jfmk10020158

**Published:** 2025-05-03

**Authors:** Elena Mainer-Pardos, Rafael Albalad-Aiguabella, Víctor Emilio Villavicencio Álvarez, Santiago Calero-Morales, Demetrio Lozano, Alberto Roso-Moliner

**Affiliations:** 1 Health Sciences Faculty, University of San Jorge, Autov A23 km 299, 50830 Villanueva de Gállego, Zaragoza, Spain; epardos@usj.es (E.M.-P.); ralbalad@usj.es (R.A.-A.); dlozano@usj.es (D.L.); aroso@usj.es (A.R.-M.); 2 Department of Human and Social Sciences, Universidad de las Fuerzas Armadas-ESPE, Quito 171103, Ecuador; victoremiliovillavicencio@gmail.com

**Keywords:** female football, asymmetry, countermovement jump, horizontal jump, injury risk, age categories

## Abstract

**Background**: Lower limb asymmetry is a critical factor influencing performance and injury risk in football players. Despite the increasing popularity of women’s football, limited research has examined how asymmetry varies across different age categories. This study aimed to investigate countermovement jump (CMJ) and horizontal jump (HJ) asymmetry in female football players across four age categories (U14, U16, U18, and +18). **Methods**: Seventy-six female football players from the same club participated in the study. Players performed unilateral CMJ and HJ tests to assess inter-limb asymmetry. A one-way ANOVA and Chi-square tests were conducted to examine age-related differences in asymmetry levels. **Results**: CMJ asymmetry significantly varied across age groups (*p* < 0.001), with the highest asymmetry observed in U14 and the lowest in +18. In contrast, HJ asymmetry remained consistently low across all age groups (*p* = 0.113). No significant correlation was found between CMJ and HJ asymmetry. Additionally, asymmetry levels in CMJ were significantly associated with age (*p* = 0.003), whereas no such association was observed for HJ. **Conclusions**: CMJ asymmetry is more prevalent in younger female football players and tends to decrease with age, suggesting that neuromuscular development plays a role in reducing asymmetry over time. These findings highlight the importance of monitoring asymmetry levels across different age groups to optimize training interventions and injury prevention strategies.

## 1. Introduction

Football is one of the most widely played sports worldwide, and in recent years, women’s football has experienced significant growth, driven by FIFA’s strategic programs [1]. This has led to a considerable increase in participation, national and international competitions, and competitive demands. Coaches and physical trainers are required to prepare their players for this new scenario, requiring them to have greater control to increase their players’ performance and minimize injury risk.

In recent years, interest in lower body asymmetry evaluation has increased among sports scientists for injury prevention, minimizing the risk of recurrence in the return to play process, and in sports performance analysis [2,3,4,5,6]. Football is a cooperation–opposition team sport in which players have to perform a wide variety of high-intensity skills that predominate because they are mainly unilateral and are determinants of sport performance, such as accelerating, sprinting, changing direction, jumping, passing, and shooting [3,7]. Previous research underscores the significance of unilateral versus bilateral strength training in enhancing these abilities and minimizing asymmetries between the lower extremities, contributing to injury prevention and overall player well-being [8,9]. Moreover, female football players exhibit a distinct injury risk profile compared to their male counterparts, with a higher incidence of concussions, knee injuries, and ankle injuries in women, whereas men have a higher incidence of hamstring and groin injuries [10].

Assessing lower limb strength in football players is commonly performed through various tests [11], highlighting the horizontal jump (HJ) and countermovement jump (CMJ) as two of the main tests to assess the explosive and elastic–explosive strength [11,12], essential capabilities for the performance of key actions in football. In addition, it has been shown that both tests are reliable and sensitive in the short term and medium term to changes induced by training in adolescent football players (for example, the CMJ demonstrated an ICC above 0.87 with a coefficient of variation below 5%, and the HJ showed an ICC above 0.90 with a CV below 3%) [13,14], making them valuable tools for monitoring physical performance progression. Both tests are also widely used in football to quantify asymmetries between the lower extremities because of their similarity to the movement patterns of football (acceleration and deceleration) and because they are similar to the dynamic stability demands of the knee during sports practice [15]. Given these attributes, these tests provide essential information for performance optimization and injury risk assessment in football players.

It is scientifically known that football injury is a complex and multifactorial phenomenon in which numerous intrinsic and extrinsic factors interact nonlinearly [16,17,18], but a recent systematic review confirmed that asymmetries between lower limbs were one of the main risk factors for injury in football [16]. Specifically, in women’s football, it is associated with a higher risk of anterior cruciate ligament (ACL) injury [19], with female players exhibiting at least twice the risk compared to male players, independent of exposure or level of participation [10]. Therefore, although inter-limb asymmetries seem to have clear support for detecting players at an increased risk of lower extremity injury [20] and for returning to sport after ACL injury [21], the influence of functional asymmetries on performance is still unclear [22], finding studies with mixed results regarding asymmetries in vertical and horizontal jumps with sprinting performance or changes of direction [7,23,24,25]. In addition, several authors in male and female football report that the performance of exercises according to the vector of force application (vertical vs. horizontal) is essential for the improvement of specific movements in these planes [12,26]. This could provide information about the training programs performed by players and the type of asymmetry they could show while presenting a solution to reduce them.

To the best of our knowledge, few studies have analyzed how lower limb asymmetries vary according to the age of the players and how they evolve throughout the maturation process, which was assessed in this study solely by chronological age. Moreno-Azze et al. [27], in their study with football players classified according to peak maturation (pre-pubertal vs. post-pubertal), reported higher asymmetries for both groups in the vertical jump compared to the horizontal jump and also found greater performance in both vertical and horizontal jumps in post-puberal players. In turn, other authors found that greater asymmetries in young male and female football players are related to lower performance in sprinting, change of direction, or jumping [23,28].

Previous research suggests that the analysis of asymmetries measured through different tests and according to age could provide useful information on the formative stage of football. Currently, it is unknown how these asymmetries vary throughout this stage. To our knowledge, no longitudinal studies have yet analyzed their evolution during the formative period. Consequently, this study aimed to explore the differences in countermovement jump (CMJ) and horizontal jump (HJ) asymmetry across age categories (U14, U16, U18, and +18) in female football players, and the possible correlations between asymmetry in CMJ and HJ, the inter-limb differences in jump performance, and the association between asymmetry levels (high vs. low) and age groups.

## 2. Materials and Methods

### 2.1. Participants

A total of 76 female football players from the same club participated in the present study. The sample was categorized into four age groups: U14 (*n* = 18, TIER 2; age: 13.07 ± 0.44 years; height: 154.09 ± 7.64 cm; body mass: 48.97 ± 7.89 kg), U16 (*n* = 18, TIER 2; age: 15.25 ± 0.39 years; height: 160.96 ± 5.15 cm; body mass: 55.04 ± 7.63 kg), U18 (*n* = 20, TIER 3; age: 17.39 ± 0.86 years; height: 155.97 ± 24.30 cm; body mass: 62.11 ± 4.31 kg), and +18 (*n* = 20, TIER 3; age: 21.00 ± 1.17 years; height: 162.41 ± 6.06 cm; body mass: 59.43 ± 4.99 kg) [29]. The TIER classification refers to the level of play, where TIER 2 includes regional-level athletes and TIER 3 corresponds to national-level athletes. These athletes had been training in football at club level for a minimum of three years, participating in three to four structured weekly technical and tactical sessions, each lasting 90 min, plus one competitive match each week. The fitness sessions varied across teams, typically including speed, agility, and quickness (SAQ) drills, injury prevention exercises, and coordination training. These sessions were planned and supervised by certified strength and conditioning coaches or professionals with equivalent qualifications, depending on the team. Their main objective was to support players’ physical maintenance and enhancement, ensuring that they met the demands of competitive match performance.

A priori power analysis was conducted using G*Power (Version 3.1.9.6, University of Düsseldorf, Düsseldorf, Germany) to determine the required sample size for statistical tests. For the one-way ANOVA comparing asymmetry across age groups (U14, U16, U18, and +18), the analysis was performed using an F-test (ANOVA: Fixed effects, omnibus, one-way). The parameters included an effect size (f) of 0.4 (large effect), α = 0.05, and power (1-β) = 0.80, with four groups. The results indicated a required total sample size of 76 players, achieving an actual power of 0.823. For the Chi-square test, which examined the association between asymmetry (high vs. low) and age groups, the analysis was conducted using a χ^2^ test (Goodness-of-fit tests: Contingency tables). The parameters included an effect size (w) of 0.4 (large effect), α = 0.05, power (1-β) = 0.80, and degrees of freedom (df) = 3. The results indicated a required total sample size of 69 players, achieving an actual power of 0.805. These results confirm that the study’s sample size was sufficient to detect meaningful effects in both analyses, ensuring the robustness of the statistical comparisons.

Participants were excluded from the study if they were undergoing medical treatment at the time or experienced pain during the jump tests. Prior to participation, all individuals received detailed information regarding the experimental procedures, as well as the potential risks and benefits associated with the study.

For participants aged 18 and over, written informed consent was obtained. In the case of minors, consent was provided by a parent or legal guardian. The study adhered to the Declaration of Helsinki (2013), with approval from the University of Zaragoza Ethics Committee (CP19/039, CEICA, Spain, approved 21 February 2019).

### 2.2. Procedures

Participants were advised to abstain from high-intensity physical activity for at least 48 h before the test to minimize the risk of muscle fatigue. In addition, they were instructed to avoid caffeine or other stimulants (e.g., energy drinks or dietary supplements) that could influence their physiological responses and overall performance.

The importance of maintaining adequate nutrition and hydration during the 48 h prior to the tests was also emphasized to ensure optimal conditions for the assessment. In addition, as all participants had previously undergone these tests on at least five occasions, they were already familiar with the procedures, which minimized any learning effect.

A warm-up protocol of the rise, activate, mobilize, and potentiate (RAMP) system was performed before the tests [30], with a total duration of approximately 15 min. Participants completed two types of unilateral jump tests, namely the unilateral CMJ and the unilateral HJ. Both tests were conducted following a standardized protocol to ensure reliability, with three attempts per leg and a 45-s rest between trials. The highest recorded value was used for analysis, as it is commonly considered to reflect the participant’s maximal neuromuscular performance capacity, and is widely used in performance testing protocols to identify peak output while minimizing the influence of submaximal efforts [31]. According to Pardos-Mainer et al. [32], both the unilateral CMJ and unilateral HJ have demonstrated excellent relative and absolute reliability in female football players. Given their high reliability, these tests were considered appropriate and were therefore used in the present study.

#### 2.2.1. Unilateral Countermovement Jump Test

The unilateral CMJ was performed using an Optojump system (Microgate, Bolzano, Italy) to assess vertical jump performance. Participants stood on one leg with their hands placed on their hips and the non-jumping leg flexed at 90° at the hip and knee. They were instructed to perform a rapid countermovement by flexing the ankle, knee, and hip joints before explosively extending the lower limb to maximize jump height. Separate trials were conducted for the left (CMJL) and right (CMJR) legs. The intraclass correlation coefficient (ICC) for this test ranged from 0.92 to 0.98, with a coefficient of variation (CV) of 3.2–4.9%, indicating excellent reliability.

#### 2.2.2. Unilateral Horizontal Jump Test

The unilateral HJ assessed the participants’ ability to generate forward propulsion. Performance was measured using a measuring tape, recording the distance covered from the take-off to the landing point. Participants began in a unilateral stance, performed a maximal horizontal jump, and were required to land on the same leg while maintaining balance. Both arm and leg swing were permitted during the propulsion phase to allow a natural jumping pattern, but only jumps where participants held the final position without losing stability were considered valid. Balance was considered to be maintained when the participant remained on the landing leg for at least 2 s without touching the ground with the contralateral foot or performing additional hops. The test demonstrated excellent reliability, with ICC values ranging from 0.90 to 0.99 and a CV of 1.9–3.4%.

### 2.3. Statistical Analysis

All statistical analyses were performed using IBM SPSS Statistics (Version 29, IBM Corp., Armonk, NY, USA). Descriptive statistics were calculated for all variables, including means and standard deviations. Normality was assessed using the Shapiro-Wilk test, and homogeneity of variances was evaluated with Levene’s test. To determine the reliability of the jump tests, the ICC and CV were calculated. To compare asymmetry in CMJ and HJ across age groups (U14, U16, U18, and +18), a one-way ANOVA was conducted for normally distributed data, followed by Tukey’s post-hoc test to identify specific between-group differences. In the case of non-normally distributed data, the Kruskal–Wallis test was applied, followed by Dunn’s post-hoc test with Bonferroni correction. To analyze differences between the stronger and weaker leg, a paired *t*-test was performed for normally distributed data, while the Wilcoxon signed-rank test was used for non-parametric comparisons. The relationship between asymmetry in CMJ and HJ was examined using Pearson’s correlation coefficient for normally distributed data and Spearman’s rank correlation coefficient for non-normal distributions. To assess the association between asymmetry CMJ and HJ levels (high vs. low) and age group, a Chi-square test (χ^2^) was performed. Post-hoc pairwise Chi-square tests were conducted between age groups. Statistical significance was set at *p* < 0.05.

Inter-limb asymmetry was calculated using the following Equation [33]:

Inter-limb asymmetry = 100/Max Value (right and left)*Min Value (right and left)*−1 + 100.

## 3. Results

Table 1 presents the descriptive statistics of CMJ and HJ performance across age groups.

**Figure 1:**
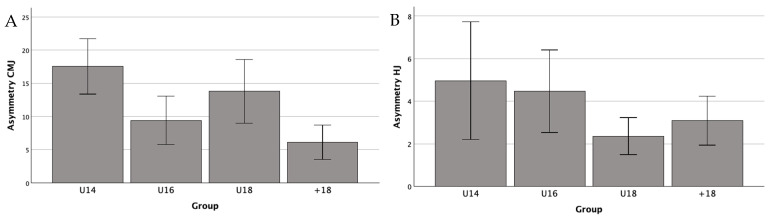
Comparison of inter-limb asymmetry in CMJ (**A**) and HJ (**B**) across age groups.

A one-way ANOVA was conducted to analyze differences in asymmetry in CMJ and HJ across age groups. The results showed a significant effect of age group on asymmetry in CMJ (F(3, 72) = 7.257, *p* < 0.001). Post-hoc Bonferroni comparisons revealed significant differences between U14 and +18 (*p* < 0.001), U16 and +18 (*p* = 0.021), and U18 and +18 (*p* = 0.022) (Figure 1A). However, no significant differences were found between groups for asymmetry in HJ (F(3, 72) = 2.06, *p* = 0.113) (Figure 1B).

A paired *t*-test was performed to analyze the differences between the stronger and weaker leg across age groups in Table 2. Results showed no statistically significant differences (*p* > 0.05) between the stronger and weaker leg for both the CMJ and HJ tests in any age group. The ES were generally small to moderate, with values ranging from 0.99 to 1.03.

**Figure 2:**
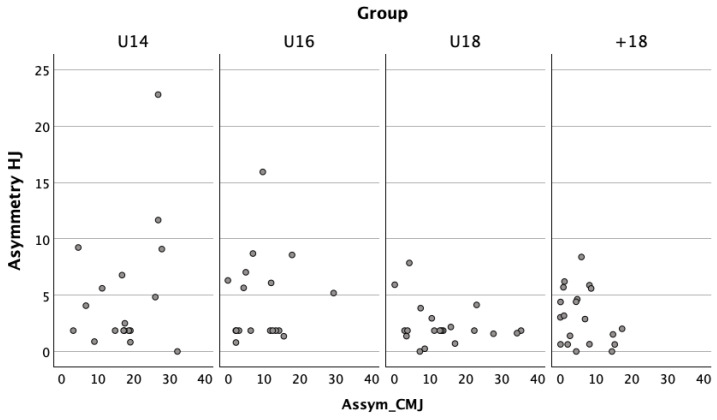
Correlation between CMJ and HJ asymmetry across age groups. Each dot represents the individual asymmetry values of a single participant.

The relationship between asymmetry in CMJ and HJ was examined using Pearson’s and Spearman’s correlation coefficient. The results in Figure 2 showed no significant correlations between CMJ and HJ asymmetry in any age group (*p* > 0.05). The correlation coefficients (r) ranged from 0.090 to −0.284, indicating weak and non-significant relationships.

The distribution of asymmetry levels in CMJ and HJ across age groups is shown in Table 3. In CMJ, the percentage of athletes classified with high asymmetry was highest in the U14 group (77.8%), followed by U18 (60.0%), U16 (44.4%), and the lowest in +18 (20.0%). Conversely, the proportion of athletes with low asymmetry increased with age, being 80.0% in +18, 55.6% in U16, 40.0% in U18, and only 22.2% in U14. In HJ, the prevalence of high asymmetry was significantly lower across all age groups compared to CMJ. The U14 group presented the highest percentage (11.1%), followed by U16 (5.6%), while no athletes in U18 or +18 exhibited high asymmetry in HJ. In contrast, the majority of participants were classified with low asymmetry, reaching 100% in U18 and +18, 94.4% in U16, and 88.9% in U14.

A Chi-square test (χ^2^) was conducted to examine the association between asymmetry levels (high vs. low) in CMJ and HJ and age groups in Table 4. The overall Chi-square test in CMJ asymmetry showed a statistically significant association (χ^2^ = 13.778, *p* = 0.003). Post-hoc pairwise comparisons revealed that the U14 vs. U16 comparison showed a moderate association (χ^2^ = 4.208, *p* = 0.040). The strongest differences were found between U14 vs. U18+ (χ^2^ = 12.865, *p* < 0.001) and U16 vs. U18 (χ^2^ = 12.865, *p* < 0.001). No significant differences were found between U14 vs. U18 (*p* = 0.239) or U16 vs. U18+ (*p* = 0.106). Fisher’s exact test confirmed significant differences in U14 vs. U18+ (*p* < 0.001), U16 vs. U18 (*p* < 0.001), and U18 vs. U18+ (*p* = 0.011). In contrast, the overall Chi-square test for HJ asymmetry did not show a statistically significant association with age groups (χ^2^ = 4.203, *p* = 0.240).

## 4. Discussion

The present study examined the differences between age categories regarding asymmetry in horizontal and vertical jump performance in adolescent female football players. The results of this study revealed that asymmetry in CMJ varied significantly across age groups, with the highest levels observed in U14 and the lowest in U18+. In contrast, no significant differences were found in HJ asymmetry between age groups. In addition, no significant differences were observed between the stronger and weaker leg in any age group for both CMJ and HJ. Correlation analysis showed no significant relationships between CMJ and HJ asymmetry in any age group, indicating that asymmetry in these tests is independent. The Chi-square test further revealed that asymmetry levels in CMJ were significantly associated with age, with the most pronounced differences found between U14 vs. +U18 and U16 vs. U18, whereas no significant association was observed between HJ asymmetry and age. These findings indicate that CMJ asymmetry is more prevalent in younger female football players and tends to decrease with age, while HJ asymmetry remains consistently low and unrelated to age.

Jump tests provide a practical and rapid means of physical assessment, often mimicking movement patterns (i.e., triple extension of the ankle, knee and hip joints) observed in sports such as jumping, sprinting, and change of direction [34,35]. Previous research has used bilateral and unilateral tests to measure asymmetries, such as the horizontal jump [7,36] and the CMJ [23,36]. Therefore, recognizing the importance of this measure and having mastered the range of protocols previously used to assess asymmetry, practitioners should consider the most appropriate test for their athletes based on analysis of sport-specific demands, players’ training history and previous familiarity with testing protocols. In summary, the assessment of asymmetries of specific muscles or muscle groups is an important measure that can help clarify the source of potential asymmetries in specific performance tasks.

In our research, greater asymmetries were observed in the vertical unilateral jump tests than in the horizontal unilateral jump tests (17.54 ± 8.36 vs. 4.97 ± 5.56), and this is in agreement with previous research carried out in young female football players [4,23]. One possible explanation for vertical jumping being more sensitive than horizontal jumping in identifying asymmetries is that horizontal jumping activities are exercised from an earlier age, learning horizontal movement patterns prior to vertical ones [23,37,38]. However, more research is needed to confirm these theories.

When analyzing asymmetries in relation to age (Table 1), it was observed that younger players exhibited higher asymmetry levels in both the CMJ (U14: 17.54 ± 8.36 vs. U18: 6.12 ± 5.56) and the HJ (U14: 4.97 ± 5.56 vs. U18: 3.09 ± 2.45). However, this difference was only statistically significant in the CMJ, whereas the HJ showed no significant differences. Furthermore, the distribution of asymmetry levels by age group (Table 3) revealed that a higher percentage of players classified with high asymmetry were in the U14 category for both the CMJ (77.8%) and the HJ (11.1%). These findings align with previous research on young female footballers, which has demonstrated that older players tend to exhibit lower asymmetry levels in jump tests [2,5,23,39,40]. One possible explanation is that female athletes typically reach a plateau in athletic performance during puberty, around the age of 13 [41], potentially leading to a reduction in functional asymmetry differences with age [42]. In addition, the Chi-square test confirmed a significant association between age and CMJ asymmetry, with the most pronounced differences observed between U14 vs. +U18 and U16 vs. U18. In contrast, no significant association was found between HJ asymmetry and age, indicating that CMJ asymmetry is more prevalent in younger players and tends to decrease with age, whereas HJ asymmetry remains consistently low and unrelated to age. This difference may be attributed to the fact that training programs appear to be more effective in reducing CMJ asymmetry, making the difference between U14 and +U18 statistically significant. Conversely, HJ asymmetry is initially lower, with a more limited margin for improvement, which could explain the lack of significance. Moreover, the unilateral CMJ requires greater neuromuscular control and postural stability, as players must manage both the landing and take-off phases using a single leg. In contrast, the unilateral HJ is less reliant on eccentric control, meaning that reduced stability does not necessarily translate into pronounced asymmetry. According to Murtagh et al. [43], such postural control coupled with quadriceps size and strength are key determinants for vertical jumps, but not for forward horizontal jumps. Studies in young athletes have shown that they have a lower hip abduction strength and a lower posteromedial–lateral stability compared to adults [44,45]. Other studies, such as that of Roso-Moliner et al. [3], analyzed the impact of the application of a 10-week neuromuscular training program in highly trained female football players which included mobility exercises, dynamic stability, anterior and upper chain strength, lumbopelvic control and change of direction, observing a greater impact on the performance of the CMJ compared to the HJ (ES = 0.23 vs ES ≤ 0.10). This same research showed that this neuromuscular program reduced vertical and horizontal jump asymmetries, having a greater effect on the CMJ (−2.37% ES = 0.02) versus the HJ (−0.87% ES = 0.01). Finally, Bettariga et al. [46] published a systematic review with meta-analysis, observing that the application of training programs involving unilateral and bilateral strength and power or sport-specific exercises (such as change of direction or unilateral jumps) led to a reduction in asymmetry. All this confirms that, as players mature and train, they improve stability and unilateral strength, reducing asymmetry in CMJ.

In Table 2, no significant differences were observed between the stronger and weaker leg in any age group for either the CMJ or the HJ. This result is particularly remarkable when compared to some of the data presented in Table 1. For instance, in Table 1, CMJ asymmetry in the U14 group is notably high (17.54%) and decreases with age. However, in Table 2, when comparing the stronger and weaker leg, the mean difference is close to zero across all groups and does not reach statistical significance. One possible explanation for this finding is the high interindividual variability within the U14 group, where some players may exhibit extreme asymmetry while others remain highly symmetrical. This variability is reflected in Table 1, which presents the average asymmetry across all players, but not in Table 2, where the comparison is made at an intraindividual level (stronger vs. weaker leg), thereby minimizing the effect of intersubject dispersion. Moreover, factors related to biological maturation could influence the manifestation of these asymmetries. Variability in somatic growth and neuromuscular changes during adolescence create significant differences in training adaptation and intermuscular coordination, which may either attenuate or amplify asymmetry depending on the player’s stage of maturation [27,47]. This phenomenon is particularly relevant when classifying young athletes, as chronological age does not always accurately reflect their true maturation status [48]. Therefore, it would be interesting to study the biological maturation of female players in future research.

Correlation analysis showed no significant relationships between asymmetry in CMJ and HJ in any age group, indicating that asymmetry in these tests is independent. From these results, a key conclusion is drawn: if practitioners wish to assess asymmetries, the use of a single test is unlikely to provide a complete picture of existing imbalances. This idea is supported by Loturco et al. [49], who demonstrated the absence of significant correlations between different frequently used unilateral jump tests. Furthermore, recent research by Bishop et al. [50] has indicated that, when comparing asymmetry scores obtained in various tests, levels of agreement tend to be low. Therefore, the findings of the present study are consistent with previous research and do not recommend the exclusive use of a single test to detect limb asymmetry. Considering that similar results have been found in various populations [49,50], it seems appropriate to suggest that the lack of association between task asymmetries is not specific to female football players, but rather an inherent and variable feature of functional asymmetry itself.

It should be noted that this study has certain limitations. Firstly, the small sample size prevented us from assessing differences between player positions. However, the main objective of this research was to examine age-category differences in relation to asymmetry, something that previous studies on male football players have done with larger sample sizes [51,52]. Secondly, due to the small sample size, we were unable to generalize the results across player positions. Nevertheless, this study focused primarily on adolescent football players’ asymmetries across different age categories regarding jump performance, rather than positional differences. Thirdly, the menstrual cycle of participants was not monitored, which may have influenced neuromuscular performance. Moreover, our findings cannot necessarily be generalized to other performance levels or team sports since the study exclusively included football players at a specific level. Despite these limitations, this research provides relevant data on assessing asymmetries among adolescent female football players and how such asymmetries influence their performance. On the other hand, future research could focus on conducting longitudinal studies based on playing position, implementing targeted interventions according to individual asymmetry profiles, or examining the correlation between these asymmetries, injury occurrence, and competitive level.

## 5. Conclusions

The present study demonstrates that younger players exhibit higher levels of asymmetry in unilateral vertical jump tests compared to unilateral horizontal jump tests, indicating that the neuromuscular demands of the CMJ might better highlight inter-limb differences. Moreover, CMJ asymmetry was significantly greater among younger players, notably decreasing with age, suggesting a progressive development of neuromuscular control and postural stability. In contrast, asymmetry levels in the HJ remained consistently low and stable regardless of age. Furthermore, given that asymmetries identified in different tests (CMJ and HJ) were unrelated, it is unlikely that a single test provides a comprehensive overview of muscular imbalances. Practitioners should therefore use multiple tests tailored specifically to the demands of the sport being evaluated. Finally, younger players (U14) exhibited greater interindividual variability in asymmetry levels, indicating that some athletes may have pronounced imbalances, whereas others could be considerably more symmetrical. These conclusions emphasize the importance of conducting multiple specific assessments to adequately detect asymmetries and highlight the need for training programs tailored to address these asymmetries effectively, taking into account athletes’ age groups and developmental stages.

## Figures and Tables

**Table 1:** Descriptive data of CMJ and HJ by age groups.

Group	CMJR	CMJL	Asym CMJ	HJR	HJL	Asym HJ
U14	10.48 ± 2.54	10.83 ± 3.29	17.54 ± 8.36	113.67 ± 9.82	114.22 ± 11.89	4.97 ± 5.56
U16	12.49 ± 2.45	12.66 ± 3.51	9.41 ± 7.35	130.61 ± 16.51	127.56 ± 11.64	4.47 ± 3.90
U18	12.35 ± 2.25	12.24 ± 2.83	13.78 ± 10.24	131.95 ± 14.26	132.58 ± 14.06	2.36 ± 1.86
+18	13.79 ± 2.21	13.87 ± 2.19	6.12 ± 5.56	143.52 ± 12.72	143.13 ± 12.43	3.09 ± 2.45

Asym: asymmetry; CMJ: countermovement jump; HJ: horizontal jump; R: right; L: left.

**Table 2:** Comparison of the asymmetry between the stronger and weaker leg in CMJ and HJ across age groups.

Group	Comparison	MD ± SD	*p*-Value	ES
U14	Stronger vs. Weaker CMJ	0.00 ± −0.46	0.5	1.03 (−0.46; 0.46)
	Stronger vs. Weaker HJ	0.17 ± −0.30	0.241	0.99 (−0.30; 0.63)
U16	Stronger vs. Weaker CMJ	0.00 ± −0.46	0.5	1.03 (−0.46; 0.46)
	Stronger vs. Weaker HJ	0.00 ± −0.46	0.5	1.03 (−0.46; 0.46)
U18	Stronger vs. Weaker CMJ	−0.10 ± 0.54	0.333	1.02 (−0.54; 0.34)
	Stronger vs. Weaker HJ	0.05 ± −0.39	0.413	1.00 (−0.39; 0.49)
+18	Stronger vs. Weaker CMJ	0.00 ± −0.44	0.5	1.03 (−0.44; 0.44)
	Stronger vs. Weaker HJ	0.10 ± −0.34	0.325	0.99 (−0.34; 0.54)

MD: mean difference; SD: standard deviation; ES: effect size; CMJ: countermovement jump; HJ: horizontal jump.

**Table 3:** Comparison of asymmetry levels in CMJ and HJ across age groups.

Variable	Group	High Asymmetry (*n*)	Low Asymmetry (*n*)	Total (*n*)	High Asymmetry (%)	Low Asymmetry (%)
CMJ	U14	14	4	18	77.8	22.2
	U16	8	10	18	44.4	55.6
	U18	12	8	20	60.0	40.0
	+18	4	16	20	20.0	80.0
HJ	U14	2	16	18	11.1	88.9
	U16	1	17	18	5.6	94.4
	U18	0	20	20	0	100
	+18	0	20	20	0	100

CMJ: countermovement jump; HJ: horizontal jump.

**Table 4:** Chi-square test results for the association between asymmetry levels in HJ and CMJ and age groups.

Comparison	Chi-Square	*p*-Value	Likelihood Ratio	Exact Fisher Test	*p*-Value (Fisher)
General HJ	4.203	0.240	—	—	—
General CMJ	13.778	0.003	9.37	—	—
U14 vs. U16	4.208	0.040	4.314	0.086	0.043
U14 vs. U18	1.386	0.239	1.408	0.307	0.205
U14 vs. U18+	12.865	<0.001	13.488	<0.001	<0.001
U16 vs. U18	12.865	<0.001	13.488	<0.001	<0.001
U16 vs. U18+	2.620	0.106	2.651	0.103	0.102
U18 vs. U18+	6.867	0.010	6.904	0.009	0.011

CMJ: countermovement jump; HJ: horizontal jump.

## Data Availability

Dataset available on request from the authors.

## Abbreviations

The following abbreviations are used in this manuscript:

HJ	Horizontal Jump
CMJ	Countermovement Jump
ACL	Anterior Cruciate Ligament
